# A framework for integrated environmental health impact assessment of systemic risks

**DOI:** 10.1186/1476-069X-7-61

**Published:** 2008-11-27

**Authors:** David J Briggs

**Affiliations:** 1 Department of Environmental Epidemiology and Public Health, Imperial College London, Norfolk Place, London, W2 1PG, UK

## Abstract

Traditional methods of risk assessment have provided good service in support of policy, mainly in relation to standard setting and regulation of hazardous chemicals or practices. In recent years, however, it has become apparent that many of the risks facing society are systemic in nature – complex risks, set within wider social, economic and environmental contexts. Reflecting this, policy-making too has become more wide-ranging in scope, more collaborative and more precautionary in approach. In order to inform such policies, more integrated methods of assessment are needed. Based on work undertaken in two large EU-funded projects (INTARESE and HEIMTSA), this paper reviews the range of approaches to assessment now in used, proposes a framework for integrated environmental health impact assessment (both as a basis for bringing together and choosing between different methods of assessment, and extending these to more complex problems), and discusses some of the challenges involved in conducting integrated assessments to support policy.

Integrated environmental health impact assessment is defined as a means of assessing health-related problems deriving from the environment, and health-related impacts of policies and other interventions that affect the environment, in ways that take account of the complexities, interdependencies and uncertainties of the real world. As such, it depends heavily on how issues are selected and framed, and implies the involvement of stakeholders both in issue-framing and design of the assessment, and to help interpret and evaluate the results. It is also a comparative process, which involves evaluating and comparing different scenarios. It consequently requires the ability to model the way in which the influences of exogenous factors, such as policies or other interventions, feed through the environment to affect health. Major challenges thus arise. Chief amongst these are the difficulties in ensuring effective stakeholder participation, in dealing with the multicausal and non-linear nature of many of the relationships between environment and health, and in taking account of adaptive and behavioural changes that characterise the systems concerned.

## Introduction

Environmental effects on health have always been multi-facetted. Even when the immediate causes have been specific and clear, and the health outcomes limited, as in the case of many natural hazards or chemical contaminants, their origins typically have had deeper and more far-reaching roots. In more recent years, however, the scale and complexity of environmental health problems have become more apparent. Many factors are behind this change [1]. One is the more powerful technologies now being used, each with the potential to change the environment more extensively and radically. Another is the increased globalisation and connectedness of societies, as a result of which impacts are not restricted to those locally and immediately involved, but are felt more extensively – in terms of economic and social effects as well as health, on people far-removed from the origin of the hazard, and even on future generations. In addition, policies themselves have become more expansive, in response to changing policy concepts, structures and imperatives. Many modern environmental threats to health are thus examples of what have been termed systemic risks [2,3]: complex risks to health embedded in wider environmental, social, economic and political systems.

As the International Risk Governance Council have argued [4], systemic risks demand more integrated and precautionary approaches to risk governance. Precaution is essential because systemic risks typically take a long time to play out, spread widely, and have long-lasting effects; early intervention is thus required to control the risks before they become established. Policies need to be more integrated both because the problems themselves are complex and interconnected, and because they cut across traditional policy-making structures, and require collaboration by different agencies, in different policy areas, at different levels of administration and different spatial scales. At the same time, both the costs of policies and the costs of getting them wrong are increasing, so demands for financial accountability and public transparency of policies have grown [5,6]. In addition, the broad scope of systemic risks inevitably means that many different stakeholders are implicated – as purveyors, victims or managers of the risks [7]. These need to be informed and involved, not only because they have a moral entitlement to know what risks confront them, but also because they are crucial agents for risk response.

In Europe, many of these principles were recognised in environmental policies at a relatively early date. Since the 1970s, for example, integration and precaution have been underpinning ideals of the Environment Action Plan of the European Union [8]. Health policies, however, have been slower to respond. Only with the adoption of the Amsterdam Treaty in 1997 did the EU agree a formal commitment to ensure (rather than merely contribute to) human health protection, and only in 2003 did the World Health Organisation's initiative to establish national environmental health action plans [9] translate into a European plan [10]. The European Environment Health Action Plan nevertheless marks an important turning point in health policy, and has significant implications for the sciences on which it depends. As well as setting out priorities for action in relation to health outcomes and key risk factors, it highlights the need for better, more timely and more integrated, information to support policy. It thus calls for work to make "assessment of the *overall *environmental impact on human health more efficient by taking into account effects such as: cocktail effects, combined exposure, and cumulative effects." It emphasises also the need for "an environment and health '*cause-effect framework*' that will provide the necessary information for the development of Community policy dealing with sources and the impact pathway of health stressors."

A number of EU-funded research projects have been established in response to this call, aimed at developing and applying new, integrated methods for assessment to support environmental health policies. This paper draws on thinking in two of these – INTARESE and HEIMTSA – to review current approaches to assessment, set out a conceptual and analytical framework for integrated environmental health impact assessment aimed at linking and enhancing current approaches, and discuss some of the challenges involved in applying it for policy support.

## Review

### Assessment in the context of risk governance

Recognition of the systemic nature of risks to human health has stimulated a vigorous debate within the policy arena about how best to develop and guide policies in the context of complexity. One of the important concepts to emerge as a result has been that of risk governance [4]. This sees risk management not as a closed (and often *post hoc*) activity, carried out by an expert elite on behalf of their policy-masters, but as a transparent and shared process amongst stakeholders. The information needed to support this process is necessarily varied. It must take account of the multiple causes and outcomes, and the multitude of intervening pathways, that characterise systemic risks. It also needs to be framed in a way that meets the demands and expectations of many different users and stakeholders, in both professional and lay roles. In addition, in order to support policy, different forms of assessment are needed. No formal typology yet exists, but three types of assessment can usefully be defined. What might be termed *diagnostic assessments *are required to determine whether a problem exists, and if so its magnitude and causes: their role is thus to help decide whether policy action is needed, and to prioritise competing demands. *Prognostic assessments *are needed to evaluate and compare the potential implications of new policies – and thus to help choose between them. *Summative assessments *have to be carried out to evaluate the effectiveness of existing policies, and provide an indication of the extent to which they are meeting their objectives: they thereby help to decide whether adjustments to prevailing policies are needed, and to inform those concerned about what is already being done.

Over the years, many approaches to assessment have been devised. In the context of health, the dominant paradigm has been that of risk assessment. Initially developed in the 1970s, within ten years this had become established in the USA as a major tool for regulation and risk management [11]. Silbergeld [12] thus defined it as "a set of decision rules ... for identifying and quantifying the risks of chemicals and other events for adverse effects to human health, usually cancer". Since then, however, risk assessment has been adopted for routine application in many countries, world-wide, and in the process its concepts and methods have diversified and changed [13], while its scope has broadened to encompass non-carcinogenic effects and a wider range of exposures, including ionising radiation, food and physical (e.g. natural) hazards [14].

Traditional forms of risk assessment have undoubtedly done good service, especially in regulatory policy fields (e.g. in setting limit values for emissions, or targets and standards for environmental quality). Risk assessment of potentially hazardous chemicals produced or marketed in the EU, for example, remains a vital (though somewhat controversial) requirement under the REACH regulation [15]. As Renn and others have argued, however, it is too narrow in focus and too unitary in approach to deal effectively with the complex inter-relationships and dynamics that characterise modern systemic risks to health [16,17]. In recent years, therefore, various attempts have been made to extend or redefine risk assessment in order to meet these changing information needs. Unfortunately, in the process, the landscape of assessment has become somewhat cluttered and confused. 'Integrated risk assessment', for example, has been promulgated in a number of different forms. While, WHO/IPCS [18] define it as a "science-based approach that combines the processes of risk estimation for humans, biota and natural resources in one assessment", it has also been described as a method to examine different hazards or agents in combination; as a means to link toxicological and epidemiological evidence; as the assessment of the overall impacts of a single chemical; or as a multi-disciplinary approach to risk assessment [19,20]. Under whatever definition, there are as yet few examples of its practical application. Bridges and Bridges [21], however, outline how it can be used to assess the risks from multiple exposures associated with endocrine disrupting agents, by considering agents that have a common mechanism or effect. Likewise, Ross and Birnbaum [22] describe its potential application to persistent organic pollutants (POPs). In these and other examples, the approach has remained agent-based, but Bonano *et al*. [23] take a somewhat broader approach, and include stakeholder participation and consideration of socio-economic, cultural and other effects to assess alternative strategies for remediation of a contaminated site.

Comparative risk assessment (CRA) has been developed in parallel. Murray *et al*. [24] define it as "a systematic evaluation of the changes in population health which would result from modifying the population distribution of exposure to a risk factor or a group of risk factors." As such, it provides a means of quantifying the contribution to the overall burden of disease from different risk factors, in a comparable and consistent way – as in assessments of the regional or global environmental burden of disease [25,26]. It can also be used prognostically or summatively to assess the disease burden from policy, as outlined by Kjellstrom *et al*. [27] in relation to transport.

Health impact assessment (HIA) provides an alternative paradigm. In contrast to risk assessment, this focuses on policies, or other interventions, rather than agents or events [28-30]. It also recognises that the environment is not just a hazard, but equally serves a beneficial role by providing natural capital [31] or ecological services [32] – for example through water security, improved nutrition or access to green space. Policies are thus concerned with enhancing the human condition through positive action to maintain, and improve access to, environmental benefits, as well as reducing risks. As such, they need to be judged in terms of the balance between potential negative and positive effects.

To date, most applications of HIA have tended to be relatively local and limited in scope – e.g. for urban regeneration schemes or local transport policies rather than broader policies [29,33]. As with many other methods of assessment, however, HIA has evolved in somewhat disparate forms and been defined in different ways [34]. For some, the focus is on stakeholder participation [29]; for others, it is primarily a means of quantification. In more quantitative assessments, the results are generally expressed in terms of the attributable change in morbidity or mortality, usually (unlike CRA) for each endpoint separately – aggregation is thus left to the user. It also relies on modelling to determine likely exposures and epidemiological knowledge to translate these into estimates of potential health effects [28,35,36]. Based on a review of 98 prospective (i.e. prognostic) assessments, however, Veerman *et al*. [37] conclude that quantification is so far relatively rare, and the validity of the methods and results uncertain. A clear danger is that assessments will be subjective, and insufficiently rigorous to provide reliable estimates of impact [34].

Similar developments have occurred independently in other policy fields. Traditional methods of environmental impact assessment (EIA), for example, applied at the level of individual projects [38], have given way to more inclusive methods of strategic environmental assessment (SEA) at the level of programmes and plans [39,40]. At the same time, concepts of integrated assessment (IA) have emerged. Initially developed in response to concerns about atmospheric acidification [41-44] and climate change [44-48], these have since been extended to a range of other environmental issues including land use change [49,50], air pollution [51-53] and catchment management [54]. As with risk and health impact assessment, IA has diversified in the process, so is now not one thing but several [46]. Rotmans and van Asselt [48] describe it as "an interdisciplinary and participatory process of combining, interpreting and communicating knowledge from diverse scientific disciplines to allow a better understanding of complex phenomena"; Rotmans and Dowlatabadi [47] note that this should be done synoptically for the full impact chain. Others, however, define it in terms of modelling and model-linkage, through which large and complex environmental systems can be analysed [52,55,56]; for these, the term integrated assessment modelling (IAM) is often preferred. Hisschemöller *et al*. [57], with some justification, argue for a combined approach. Inherently, however, IA takes an explicitly systems-based approach and as such can perhaps better (and more simply) be defined as the systemic analysis of complex societal problems, as a basis for policy support.

IA clearly provides a valuable tool for analysis of environmental issues, and has already been adopted by the European Environment Agency both for assessment of, and reporting on, the state of the environment [58]. It thus, also, offers a potentially valuable, integrating paradigm for assessment of policies affecting human health. Nevertheless, even though many of the issues to which IA has been applied clearly have direct relevance to human health, surprisingly few attempts have yet been made to extend assessments to health [43,53,59]. Nor has IA been taken up by environmental health scientists, despite the repeated call to do so, especially in relation to problems such as climate change and infectious diseases [60-62]. The need to develop a more inclusive and integrated approach to assessment of environmental health risks and policies thus remains.

### Integrated environmental health impact assessment: a conceptual framework

**Figure 1:**
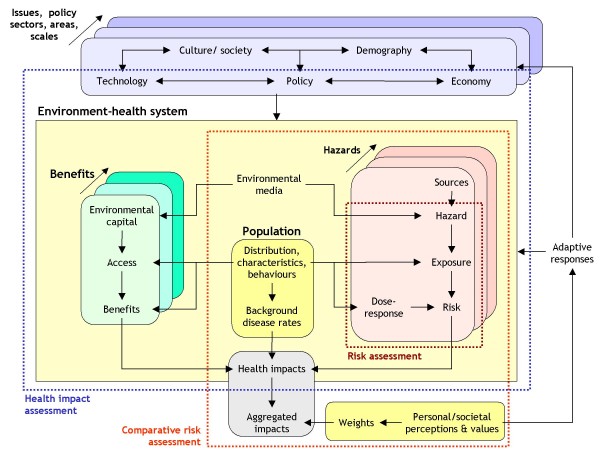
Integrated environmental health impact assessment in relation to other forms of risk and impact assessment.

As already noted, the various methods of risk and health impact assessment developed over recent years have resulted in a somewhat confused situation. Figure 1 both illustrates the problem and suggests a solution. The problem lies in the existence of a number of overlapping approaches that nevertheless fail to meet the needs of policy-makers for an integrated methodology for assessment [10,19,60,63] – a function in part of the differences in scientific perspective, inconsistencies in concept and somewhat lax use of terminology that have pervaded much of the research on assessment in the past. The solution offered in Figure 1 is to provide a framework for what is termed here *integrated environmental health impact assessment*. The purpose of this is twofold: first, to bring together existing methods within a more coherent system, so that users can choose more sensibly between them; secondly, to extend these methods in order to provide a more comprehensive methodology for assessing complex, systemic risks and policies.

The logic underlying this framework is as follows. Integrated environmental health impact assessment is defined as a means of assessing health-related problems deriving from the environment, and health-related impacts of policies and other interventions that affect the environment, in ways that take account of the complexities, interdependencies and uncertainties of the real world. As such, it takes a broad and inclusive concept of both the environment and health. In terms of the environment, for example, it covers not only environmental hazards, such as chemical hazards or environmental contaminants, that have traditionally been the focus of risk assessment, but also any other aspect of the ambient and living environment that may affect health, either negatively or positively. Health, equally, is seen not only in terms of morbidity and mortality, but more widely in terms of human well-being. Effects on health thus operate either via human exposures to environmental hazards, or by human access to and exploitation of environmental capital and services. Both are mediated via human behaviours and perceptions, and as such are a function of where people live and spend their time, the personal and societal characteristics of the populations (age, gender, socio-economic status, culture, belief-systems etc) and the associated susceptibilities, attitudes and values.

The whole environmental health system is, in turn, subject to exogenous influences. These derive from a wide range of factors, including not only policies but also other interventions and developments, such as technological, socio-demographic and economic changes. These external factors act as forces for change *within *the system: for example, by altering the state of the environmental capital or hazards, by influencing population distribution, characteristics and behaviours, and by affecting health care and other factors (e.g. awareness raising, insurance systems) that condition their impacts. Integrated environmental health impact assessment, therefore, involves analysing the impacts of the environmental capital and hazards within the context of these changing external forces. Likewise, impacts of change can feed back to affect these drivers – for example, by influencing social and demographic structures or economic conditions, or by leading to further policy initiatives.

Integration within the framework thus occurs in several different dimensions: distally, along the full length of the causal chain from remote sources to ultimate health effects; laterally, across different sources, risk factors, pathways of propagation and health outcomes; sectorally, across different policy areas and administrations; geographically, across different regions and spatial scales; and temporally over different time dimensions from past to future, and short- to long-term. The framework also explicitly incorporates and links traditional risk assessment and CRA, as well as HIA. As in IA, however, it expands these to cover more complex, multi-sectoral issues and policies.

### The assessment process

**Figure 2:**
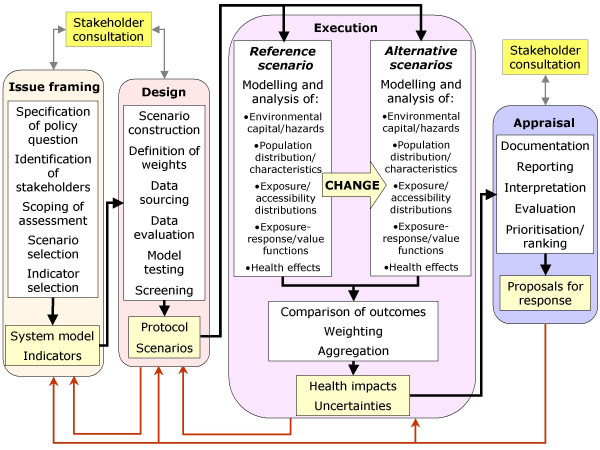
An operational framework for integrated environmental health impact assessment.

This concept of integrated assessment for policy on environment and health has inevitable implications for the way in which assessment is carried out, and who is involved in the process. Compared to the simple three- or four-step sequence commonly used to describe traditional risk assessment – i.e. hazard identification, dose-response assessment, exposure assessment and risk characterisation [13,14] – the process of integrated environmental health impact assessment is much extended. In particular, much more attention needs to be focused on the earlier stages of analysis – what IRGC [4] termed the pre-assessment stage – in order to ensure both that that the issue has been fully defined and agreed, and that an appropriate form of assessment has been chosen. In addition, effort needs to be given to the interpretation and evaluation of the results, to make sure that they are properly understood and accepted by the stakeholders involved. As such, assessment extends also into what IRGC [4] term 'risk evaluation'. Figure 2 thus defines a four-stage procedure, comprising issue-framing, design, execution and appraisal; and because such frameworks seem to require an acronym, we can thus call it the IDEA process.

#### Issue framing

The process starts with issue-framing. This stage is crucial, for the way in which the problem (and the purpose of the assessment) is defined inevitably focuses and limits the scope of the assessment, and the management options that are considered. Its purpose, therefore, is not only to set out clearly and unequivocally the policy question or problem that needs to be addressed, but also to agree the scope of the assessment – to set the boundaries of the problem, decide what elements of it are important and what are not (including whose interests should be taken into account), and to outline the policy scenarios that should be considered. In general terms, therefore, issue framing involves constructing a conceptual model of the issue to be addressed, which thence provides a framework for the assessment.

This is, by definition, a discursive process. Accordingly, it needs the participation not only of the scientists responsible for assessment, but also its users (e.g. policy-makers) and other stakeholders on whose behalf the assessment is done. This can be a difficult process, because of the inevitable differences in understanding and power of those concerned. A range of techniques is nevertheless available for this purpose. Simple textual narratives, for example, provide a rich means of defining issues [64], though graphical methods may be preferred for the sake of clarity, to avoid some of the ambiguities inherent in words, and to give more structure to the model. Mindmaps are powerful and flexible techniques to visualise people's perceptions of problems, and are especially useful in dealing with lay stakeholders – though the resulting maps may need to be recast to translate them into an organised and logical representation of the system [65]. System diagrams, as widely used in environmental sciences and engineering [66], provide a more rigorous means of conceptualising complex problems on the basis of their inherent functional or ecological structure. Causal diagrams and directed acyclic graphs (DAGs) are now gaining in favour in epidemiology [67-69] and have the advantage of enforcing a yet stricter logic on model-building.

By whatever means it is done, issue-framing will rarely be a simple linear process, but instead is likely to involve a (sometimes repeated) cycle of complexification, as additional elements and links are incorporated in response to new interests or insights, and simplification, as attempts are made to eliminate less important or irrelevant components and make the assessment more practicable. For this reason, it is essential to agree in advance both 'rules for engagement' and a clear structure for issue framing. Perhaps the most appropriate such structure in the context of integrated assessment is the impact pathway: the chain of causality that links original sources with health outcomes and their consequences. Many frameworks depicting this pathway have been developed in recent years, primarily for the purpose of indicator development [70-77]. Amongst these the so-called DPSIR framework (driving forces-pressures-state-impacts-responses) has been widely adopted for environmental assessment and reporting [58,78]. The similar DPSEEA framework [79,80] substitutes the last two links in this chain with exposures, health effects and actions and has likewise been extensively applied as a basis for developing environmental health indicators [70-72]. Both these frameworks have been criticised as being too linear, and various alternatives have been proposed, emphasising the importance of contextual factors as determinants of exposure, susceptibility and health impact [73-77]. In practice, any one of these frameworks may be applicable, and the choice between them (or the decision to devise a purpose-designed framework) will depend in part upon the issue under consideration and at which link within the chain attention is addressed. The key requirement is to use a framework which, on the one hand, captures the multivariate nature and the full extent of the impact pathway, so that important factors are not ignored, and on the other provides an understandable organising structure for those involved.

#### Design

The purpose of the design stage is to convert the conceptual model devised during issue framing into a detailed protocol for assessment. Often this will involve reconfiguring the initial representation of the system into a more organised structure, that better matches the analytical procedures that need to be undertaken – e.g. by defining clearly the key variables and their relationships, the directions of effect, relevant contextual or confounding factors, and the specific metrics that will be computed during the assessment.

As part of this, also, the policy scenarios on which assessment will be based need to be specified. These are vital, for assessment is, by definition, a comparative process: it involves comparing outcomes resulting from one state with those from one or more others. The nature of the comparisons varies. In diagnostic assessments, such as environmental burden of disease studies, the comparison will typically be between the current situation, and some alternative, counterfactual state [24]. Commonly, although not always plausibly, the counterfactual condition is defined as the total absence of the risk factor(s) under consideration – e.g. zero pollution levels. More realistically, it may be set in terms of a policy standard or a supposedly tolerable level of risk. For prognostic assessments, the comparison is between a base case, typically represented by the *status quo *or, better, some projection of it under a business-as-usual scenario, and one or more alternative (e.g. policy) scenarios. For summative assessments, the comparison is between the current state and that at some previous, pre-intervention time (or a business-as-usual extrapolation from it to the current time).

In much traditional risk assessment the role of scenarios has often been implicit rather than explicit. Where they have been defined at all, scenarios have tended to be simple and prescriptive, representing static exposure distributions for a fixed reference population. They have thus tended to assume the achievement of a specified situation (e.g. a supposedly worst-case scenario or desired target state), without considering how this will be attained or the ancillary changes in the system that may occur in the process. This concept of scenarios is limited for it neglects the fact that most systems do not switch automatically and instantly from one state to another, but instead progressively transform through a complex course of adaptation and adjustment, as the consequences of external perturbations work through the system – until (in principle at least) it regains a new equilibrium state. Some of these adaptations may act to amplify the initial changes, while others may reduce them. In many instances, the scale of these adaptive and ancillary changes will be at least as great as – and may dominate over – the more direct and intentional consequences of the intervention. This has been well illustrated in relation to climate change, where not just temperatures but almost every aspect of the climate system, and many different aspects of natural and managed ecosystems and hydrology – along with human behaviour, economic activity, and thus population distribution, susceptibility, attitudes and value systems – may all change [81-83]. Even a more local intervention, such as the congestion charge zone in London, may have far-reaching effects: via the cost of road transport within the zone, to vehicle numbers, the make-up of the vehicle fleet, journey times and durations, route choice, speeds and demand for other transport modes; and from these in turn to shopping, leisure and work patterns, and thence to commercial property and house prices – and potentially beyond that to the social makeup of the local population. Failure to recognise these adaptive responses will almost invariably lead to partial and biased assessments of policy impacts.

A further and important step in design is screening. One aim of this is to determine whether, and how, the assessment should proceed. Not all issues merit a full, integrated assessment, and some may not be worth assessing at all. Some scenarios, for example, may imply no significant changes in risk to, or the quality of, human health: the changes in the environment may be inherently neutral in terms of health, or existing regulations (or adaptive responses) may prevent significant exposures and thereby break the link between policy and health impact. Screening can thus help to avoid carrying out unnecessary or uninformative assessments. Equally, it can help to select the most appropriate means of assessment. In some cases, existing data may be inadequate for quantitative analysis, and instead only a simpler qualitative assessment may be feasible. Traditional, unitary methods of risk assessment, focusing on the more direct risks associated with specific hazards, might be deemed appropriate where the issue is simple and the question essentially diagnostic. Comparative risk assessments may be done where the need is to estimate and compare the contributions from different risk factors. Health impact assessment may be selected where the impacts are specific and localised. An integrated assessment will thus only be appropriate where the issue merits a more complex analysis of the full causal chain. To allow such choices to be made, screening will often involve a 'first-pass approximation' of the impacts. This might be done either qualitatively (e.g. via expert assessment) or semi-quantitatively (e.g. using simple screening models). Simulation can also be a powerful method for screening: for example, to predict how the system might change, and the resulting scale of effects, under the extremes of the selected scenarios.

#### Execution

The execution stage comprises the heart of the assessment process. Like traditional risk assessment, it includes the steps of hazard identification, exposure assessment and risk characterisation [13,14,84]. As a form of health impact assessment, however, it extends this to incorporate estimation of health consequences [85], and then, as in CRA, further combines these into an overall assessment of impact through some means of aggregation. In dealing with systemic issues, the whole process is also highly multivariate, and involves analysing the combined effects of different environmental factors, operating via different pathways on different health outcomes.

As such, execution relies on the use of models, especially for exposure assessment. These are essential for two reasons. First, comprehensive, representative, measured data are rarely if ever available, so modelling is needed to fill the data gaps. Second, for most assessments there is a need either to predict future conditions or to reconstruct past (or hypothetical) ones, and this can only be done through some form of modelling. Models are, however, inevitably an approximation of reality, so validation and verification are crucial if their results are to be convincing. In practice, rigorous validation of models represents major difficulties [86,87], since the very purpose of modelling is to obtain estimates of conditions beyond the available observational data (including into the future): some degree of extrapolation into the unknown is therefore inevitable. In many cases, also, integrated assessment involves the linkage of models which, however well tested in isolation, may still pass on major and unseen uncertainties from one to another. Moreover, a close fit between modelled estimates and observations does not give proof of the validity of the model assumptions – merely its predictive ability. Such comparisons can, however, provide some check on the plausibility of model results, while sensitivity analyses offer the means to assess model behaviour in the absence of independent measurements [88]. Certainly modelling in the absence of such reality checks should be treated with suspicion.

Estimation of the health impacts of exposure to hazards, or the access of environmental capital, relies also on information on the exposure-response (or use-benefit) relationships. Traditional risk assessment has tended to use data from toxicological research for this purpose. In the case of integrated environmental health impact assessment, however, the utility of these data is more limited. Relationships derived from toxicological studies, for example, are often based on animal models, and large uncertainties arise when these are translated to humans. They are also often expressed in terms of a no-observable effect level, or similar threshold value, rather than as a continuous function. In addition, toxicological studies tend to give only limited information on variations in risk across the population. For these reasons, health impact assessments typically make more use of epidemiological studies. These, too, have their limitations. Many epidemiological studies, for example, do not provide continuous exposure-response functions, but measures of relative risk based on simpler contrasts between exposed and unexposed groups. These are rarely suitable as a basis for estimating changes in health impacts due to incremental changes in exposure distributions, without some form of further modelling or interpolation. Marked inconsistencies in the reported functions also occur – a result in part of differences in study designs (e.g. exposure metrics, models, study populations, specific health outcomes), as well as natural variation in the phenomena themselves. In order to derive best estimates of exposure-response functions from past studies, therefore, some form of systematic review is usually necessary [89]. Ideally, this should enable different studies to be weighted not only according to their quality (e.g. based on the sample size and accuracy of exposure estimation), but also their relevance to the population and area under consideration. A more fundamental problem is the simple lack of exposure-response functions for many risk factors. Equally, very little is known about the effects of combined exposures, either of pollutant species with similar modes of action in complex exposure mixtures, or of different agents (such as air pollution and noise) acting in tandem. In these cases, it may be necessary to use more qualitative methods such as expert elicitation to obtain exposure-response functions for use in integrated assessments, for example through the use of Delphi surveys or nominal group techniques [90]. However they are chosen, it is also important to recognise that the choice of exposure-response function is critical to the way in which assessment is carried out, for exposures need to be estimated in a form that is compatible with the chosen function.

Outcomes of the assessment will usually be presented as measures of impact. The indicators used for this purpose should be selected at the issue-framing stage, and may differ substantially depending both on the nature of the analysis and the stakeholders or users concerned. In summative assessments, use if often made of performance-based metrics, such as distance from target, or acceptability-related indicators (e.g. based on public opinions). These may nevertheless give only a partial indication of the true effects because the health implications of policies often extend beyond their intended consequences. In diagnostic and prognostic assessments, outcomes will usually be expressed in terms of health impact, but if these are to be directly compared (e.g. between different policy scenarios), they need to be expressed in a consistent form. Commonly used metrics such as total mortality or morbidity are therefore not always appropriate, for not all deaths are equal in terms of their timing, nor all illnesses in terms of their severity or duration. For these reasons, measures such as years of life lost (YLL), disability-adjusted life years (DALYs) or quality-adjusted life years (QALYs) have been variously proposed [91-93], while economic measures may also be applied, as in cost-benefit analysis [94]. In each case, however, the derivation of the weights (and, for long-term effects, discount rates for present versus future impact) have particular importance, for they greatly influence the outcome. Since there is no absolute truth in terms of these value judgments, the primary aim is usually to obtain weights that are both relevant and acceptable to the stakeholders involved; for this reason, selection of weights should ideally be a participatory process.

#### Appraisal

Appraisal represents the final stage in assessment, and provides the point at which results are synthesised and interpreted. It thus involves weighing and evaluation of the outcome measures for the various scenarios and, where relevant, ranking the different policy options in terms of their acceptability or effectiveness. Like the initial issue-farming, if it is to be effective, it needs to be a process of discourse during which the stakeholders, on whose behalf the assessment has been done, can express their views on the results of the assessment and their implications for action. It should also provide closure to the whole assessment process by linking the results back to the original goals defined in issue-framing, and thereby help to ensure that those involved accept the outcomes.

### The added value of integrated environmental health impact assessment

The approach to integrated environmental health impact assessment offered here clearly represents a major extension from traditional forms of risk assessment, and even from more holistic approaches such as HIA and CRA. As already emphasised, it is not intended to replace these methods: indeed many (probably the majority) of issues can be adequately addressed by these methods, without recourse to some form of integrated assessment. Where a full, integrated assessment is deemed necessary, however, considerable additional scientific and practical challenges and costs are implied: for example, in sourcing and collecting the necessary data, in modelling, and in consultation with users and other stakeholders. If these costs are to be justified, then benefits in terms of the speed, cost or effectiveness of decision-making need to be shown.

Providing such evidence is, in practice, difficult. The benefits attributable to complex interventions such as policies can only rarely be directly observed. Instead, all that can be done is to estimate the effects by some sort of cost-benefit analysis either in advance of, or following, policy implementation, as Krewitt *et al*. [43] has done for air pollution abatement targets in Europe and Levy *et al*. [95] for ozone reduction. As the latter note, major uncertainties are involved. Evaluating the costs and benefits of the assessments that led to these decisions (as opposed to the policies themselves) is more tenuous still, and involves a yet further set of assumptions about the actions that would have been taken in the absence of the assessment (or in response to a different one). Since different types of information carry different weight with decision-makers, and the translation of information into action is neither seamless nor direct, these assumptions are highly tentative. Moreover, the impacts of adopting the policy (and thus of having done the assessment) are integral parts of the original assessment, so the whole process becomes essentially tautological in using the results of the assessment to evaluate its benefits!

In addition, while several studies have illustrated the potential for integrated assessment of health impacts [27,60], there have as yet been few practical applications, so evidence on which to base such evaluations is scarce. The environmental burden of disease studies conducted both globally and in Europe [25,26] have, however, had considerable influence on health policy, and clearly demonstrate the value of broad and integrated diagnostic assessments. Wang and Mauzurell [53] also provide an informative example of using integrated assessment to estimate health impacts of air pollution under different energy policy scenarios in the city of Zaozhuang in China. Although only partial – in that it considers health impacts solely from the local effects of air pollution (and ignores wider regional impacts), and takes no account of behavioural changes in response to the policies – it illustrates the level of modelling required and the uncertainties involved. In the face of rapidly rising demand for energy, it also demonstrates the considerable health benefits and associated cost savings (up to $1.4 billion) that could be gained from improved emission controls and combustion technologies – though notably, these still fail to avoid a rise in air pollution-related deaths and morbidity. van Lieshout *et al*. [96] likewise estimate the population impact of malaria under different climate change scenarios, which implies an increase in the population at risk of up to 200 million people worldwide by 2080. Such effects, of course, represent only a small subset of the overall health impacts of climate change, but indicate the scale of effort that will be needed to control the burden of disease from malaria. These examples hint at the value to decision-making that such assessments may have.

The dangers of *not *carrying out integrated assessments of complex issues are also evident. The case of biofuels provides a clear example. Over the last ten years, policies to expand their use have been widely introduced, largely in an attempt to reduce emissions of greenhouse gases [97]. A serious but unintended consequence has been to take land out of food production, thereby contributing to raised world food prices and food shortages, and threatening the lives of millions [98]. As a result, pressures are now emerging to reverse policies promoting biofuels. The failure to recognise these adverse effects during policy development, despite clear warnings about their consequences [99], can be attributed in part to the risk-based focus of the policy debate. The aim was to find policy solutions to the downstream problem of climate change, rather than to assess the potential impacts of upstream actions (energy policies). As such, this example illustrates the importance of carrying out prognostic, as well as diagnostic, assessments as part of the process of risk governance. Many humanitarian problems are likewise systemic in nature. Though they often emerge as acute and unexpected events, their roots generally lie in the social, economic, political and environmental problems that characterise the areas concerned, and the vulnerability of the local populations [100]. In the light of these slow-acting and chronic causes, ample opportunity for early warning and preventive action should exist. That these opportunities are not taken owes much to the failure to assess the problems adequately, and to recognise the humanitarian and financial costs of acting too late. This is not to say that assessment alone would ensure earlier intervention – there are many other reasons for policy inaction – but early and integrated assessment is an essential prerequisite for early and effective response [101].

### Limitations and challenges

The framework for integrated environmental health impact assessment outlined here combines two main methodologies: an essentially qualitative approach for framing issues and selecting and designing appropriate methods of assessment; and a quantitative approach for carrying out integrated assessments of complex, systemic problems. Neither of these is without its limitations and challenges. The former is by definition a strongly participatory process, and thus has to cope with the difficulties inherent in stakeholder involvement; the latter implies the ability to model and analyse complex, multivariate systems, often in the context of limited and incomplete knowledge and data.

#### Stakeholder participation

The importance of involving stakeholders in assessment has already been made. In this context, assessment serves a triple purpose. For risk managers it provides a basis for defining, prioritising and selecting appropriate policies (or other actions). It also provides the information which needs to be communicated to other stakeholders (albeit often in a more synoptic form), in order to explain the nature of the risks, justify the actions being taken and provide an indication of what others may do to reduce the risks. Thirdly, and crucially, the process of developing the assessment provides the means to engage stakeholders in the overall process of risk governance, and thereby helps to gain their cooperation and trust. Stakeholders therefore need to be actively engaged not only in issue-framing, but also in the design stage (e.g. in selection of outcome indicators and associated weights) and during appraisal.

The demand for stakeholder participation is, of course, easy to make. Its practical implementation poses much greater problems. Certainly one of the key lessons from risk communication generally has been that stakeholders can rarely be defined, approached and effectively engaged at short notice; participation requires mutual understanding and trust, and both of these take time to build [7]. This implies developing early links with stakeholders, and being prepared to engage them in a repeated and continuous dialogue. Time and resources are thus often important barriers, especially where policy-makers need decisions in a hurry [34]. Likewise, stakeholder engagement may be hindered by suspicion of the experts and the dominance of proceduralised cultures within the authorities concerned [102]. When attempts are made to involve them, considerable difficulties can be encountered in trying to identify genuine stakeholders [7], or in finding individuals who can legitimately represent large and diverse stakeholder groups. Enabling stakeholder involvement can be equally problematic due to the differing levels of knowledge, articulacy, opportunity and power that may exist [103,104]. All these problems are severe enough in local contexts; in the case of complex regional or global issues they are greatly compounded. Methods are beginning to emerge that would enable participation of the wider public in broader decision-making – for example citizens panels and interactive websites – but any form of direct and representative stakeholder participation (as opposed to involvement by proxy via elected governments or NGOs) in major regional or global issues has so far been something of a chimera, and often ill-informed [105].

#### Multicausality

The complexity of systemic issues inevitably poses major challenges for assessment. A crucial aspect of this complexity relates to the multi-causality inherent in systemic risks. This takes different forms. Probably the most obvious and simple is in the action of different agents, as part of an exposure mixture. Where these agents have a common mode of action, their effects are likely to be simply additive, but where the components of the mixture have different characteristics and modes of action, non-additive (including synergistic and antagonistic effects) are likely to occur [105-107], making system behaviour non-linear and somewhat less predictable. Multi-causality may also arise, however, where fundamentally different agents conspire to affect health – as in the case of air pollution and noise [108] or, more contentiously, atmospheric particles and electro-magnetic radiation [109]. Equally, non-environmental factors, such as genetic characteristics or personal behaviours may act as conspirators within the causal system, as in the interactions between radon and smoking [110] in relation to cancer. Nor does multiple causation occur only through contemporaneous exposures to different agents. It may also be a result of exposures at different times of life, either to the same agent or to different ones, operating to cause either predispositional (e.g. sensitisation) or protective effects. Prenatal exposures to air pollutants and other environmental contaminants, for example, have been shown to contribute to low birthweight [111,112], which may in turn predispose people to a range of illnesses later in life [113,114]; early exposure to allergens may act either to sensitise to, or protect against, risks of respiratory illness [115].

This complex, multilayered (and multi-temporal) structure of causality has important implications for assessment. Perhaps most fundamentally, it raises questions about the very definition of causes. These, it is evident, are not restricted simply to proximal determinants of health effect, but represent a potentially endless succession of cause, back through time to ever earlier antecedent events. Moreover, causes at any step in this sequence are conditional upon other, interacting or contributory influences. On this basis, Rothman and Greenland [116] define a cause as any antecedent event, condition or characteristic that was necessary, when all other causal factors are fixed, for the occurrence of the health outcome at the time it actually occurred. Following from this Rutter *et al*. [117] argue: "Not only is there not just one cause, but none is the basic cause......there is no point in seeking to identify 'the' cause of a multifactorial disorder because there is no such thing."

In epidemiology and toxicology, the response to this situation has generally been to control for other risk factors, either through the study design, or in the statistical analysis. In assessment, this approach is not adequate, for the ultimate need is to model the complete causal system, insofar as it relates to the policy and health outcomes of interest. The nature of the interplay between different causal factors thus becomes crucial. Nevertheless, the question of how to apportion causality between two or more interacting factors remains. In many situations, such interactions may have limited, or at least rather subtle, effects. Extreme cases are likely to arise, however, where one factor is wholly conditional upon another, as perhaps in some gene-environment interactions. In these situations, the simple test of distributing the disease burden to each factor according to the proportional change in outcome achieved when controlling for one factor at a time will thus, inevitably, result in the full burden of effect being assigned to each. As a consequence, the sum of the attributed effects may far exceed 100%.

The solution to this dilemma is not easy to find, and cannot be defined simply in terms of standards of evidence such as the widely used (but perhaps much abused) Bradford Hill criteria [118] – especially as these tend to ignore many of the important biases that may affect published science [119]. Simply excluding factors that cannot readily be quantified, for example, does not in reality reduce the uncertainty in the analysis, but merely shifts the analysis towards a more narrow, and probably biased, conceptualisation of the problem, and in so doing hides the inherent uncertainties within the issue-framing process. Trying to incorporate every conceivable element of the problem, on the other hand, may make the assessment process cumbersome (and potentially unsolvable), without necessarily improving the quality of the results. How to combine qualitative and quantitative information within the assessment, given their different uncertainty structures, also remains a major challenge [120]. The answer thus has to lie in trying to optimise the assessment in relation to its purpose. As such, three factors have to be taken into account: the needs of the users, including the degree of tolerance or uncertainty they are willing to accept; the uncertainty structure within the analysis (i.e. where and to what degree uncertainties arise); and the costs of acquiring additional information. Systematic methods for defining this balance do, ostensibly, exist – for example in the form of value of information analysis [121]. These, however, depend on being able to model the error structure in the data that have not yet been included (as well as those that have), which inevitably poses difficulties. As yet, therefore, there are few examples of their application, and their practicability in the realm of complex assessments remains uncertain. As in other aspects of assessment, therefore, the main defence lies in ensuring that the procedures used for effect attribution are both transparent and logical. One principle should clearly be that summation of effects must never exceed the total disease burden available – though in some cases this may involve somewhat arbitrary division of effects between contingent factors. It should always exclude summation of effects *along *a causal pathway (i.e. of factors that lie in sequence on the same pathway). To ensure this, prior construction of an explicit causal model is essential, defining at which stages in each causal pathway summation is appropriate, in relation to the questions being addressed.

A further implication of the multi-causal systems outlined above – and a further complication in the process of effect attribution – is that health impacts cannot be explained fully by the negative, risk factors involved, but depend also on the effects of positive factors, acting either to suppress illness by inhibiting the function of the risk factors involved, or actively to improve human health. The concept of causation, in this context, thus needs to be broadened; it has to be seen not in terms of the generation or triggering of disease, but in terms of agency or instrumentality with regard to health status. In most studies of the burden of disease, the contribution of these health-positive factors is ignored – to some degree logically, since the focus is on disease burden. Excluding them may nevertheless skew perceptions of the important and counter-balancing role played by some apparent risk factors, with the further danger of encouraging inappropriate policy actions. As Lucas and McMichael [122] acknowledge, therefore, causation is an interpretation, not an entity.

#### Non-linearity

As already implied, systemic risks tend to behave non-linearly. Evidence for lower thresholds for exposure, below which adverse health effects do not occur, has often been claimed, for example, in relation to cancer [123,124]. Studies of air pollution and respiratory illness have suggested S- or inverted J-shaped dose-response functions, with reduced slopes for dose-response curves at higher levels of exposure for at least some exposures and health outcomes [125,126]. In some cases, hormetic associations have been described, characterised by U- or J-shaped dose-response functions, which appear to indicate beneficial or protective effects at low levels of exposure [127,128]. The existence of, and explanation for, such non-linear exposure-response relationships has nevertheless remained the subject of considerable dispute, not least because the presence of thresholds or other forms of non-linearity may be masked in many epidemiological study designs [129,130]. At the individual level, the existence of a lower threshold is perhaps not surprising, on the simple assumption that people are able to absorb a limited dose without detectable adverse effects. At the population level, S-shaped relationships between exposure and health effect may likewise be expected, on the assumption of a quasi-normal distribution in susceptibility across the population, such that strongest effects appear to occur at moderate exposure levels. In part, also, non-linearity may reflect the adaptive nature of the processes involved. Risk avoidance strategies, in particular, may result in markedly non-linear associations, as people who perceive themselves at risk, or who are more sensitive to the exposure, take actions to reduce their exposures or susceptibility to its effects. Such responses are evident in relation to natural disasters, for example, where mitigation strategies have been reported, including measures to damage-proof the home, home and health insurance, preparation of means of escape, and migration out of the area [131,132]. Changes in behaviour, such as sleeping in different rooms, closing windows, installing double-glazing or – *in extremis *– moving house, may also be adopted by people exposed to high levels of night-time road or aircraft noise [133,134].

For whatever reason non-linearity arises, it poses important challenges for integrated environmental health impact assessment, for it means that the magnitude of the effects is highly sensitive to the absolute levels of exposure and the specific susceptibility of the population concerned. Uncertainties in exposure assessment may thus feed through into even larger uncertainties in terms of impact, while effects of intervention are likely to depend on where within the dose-response function the system currently lies, and to where it will move. Differences in the susceptibility of the exposed population may also substantially change the true degree of impact, not always in the way expected. Moreover, non-linearity is not restricted to exposure-response relationships; it may also be anticipated in other parts of the systems under investigation. In different ways, for example, it occurs in the relationships between vehicle speed and emission rate [135], wind speed and air pollution concentration [136], distance from source and EMF power density [137], and perhaps in host population density and rate of disease transmission [138]. Indeed, it can be argued that non-linearity is the norm, rather than the exception, in many natural systems [139]. For all these reasons, simple extrapolation of future from past trends, or assumptions or proportionality in response to change, are likely to be unreliable. Equally, direct translation of results of an assessment from one area, or one policy context, to another is inherently dangerous. Instead, assessments have to be conditioned to the specific context under consideration.

#### Change, adaptation and time

As has been noted, assessment involves comparing health outcomes under different scenarios. As also noted, the transition from one state to another rarely occurs spontaneously, but usually involves considerable adaptive changes. In recent literature on, for example, climate change, discussion about adaptation has tended to focus on deliberate institutional responses to risk, primarily through policy. More generally, however, adaptation involves a complex and recursive process of individual response, not only to the risks themselves but also to the resulting policies or other interventions [7]. These collective and individual adaptations may take a long time to manifest themselves, both because of in-built latencies within the system (e.g. the lag between exposure and expression of a health outcome), and because the initial perturbation may trigger a long chain reaction of response as the effects spread out through the wider system. Adjustments may thus continue for many years or even centuries. Nor are the states at the start and end of this process necessarily some form of stasis or equilibrium, both because internal dynamics (such as ageing or evolution) may mean that change is inherent, and because other externally driven perturbations may disrupt the system before it can become fully adjusted. Many systems thus remain in a state of perpetual flux.

The dynamics of environmental health systems have important implications for assessment, for they mean that the results are dependent on the timeframe used. Comparisons of simple snapshots in time, representing before and after conditions, for example, are likely to underestimate the true impacts because they fail to take account of the (possibly substantial) effects of intervening adaptations. Assessments based on short timeframes are also likely to be misleading, for they will ignore the longer-term consequences both of the initial intervention and subsequent adaptive responses. Even so-called life cycle approaches, in which assessment is continued for the duration of the policy or product, may neglect more persisting legacies, such as inter-generational effects. If these long-lasting effects are to be considered, the timeframes for assessment may need to be extremely (and somewhat arbitrarily) extended. In a life cycle analysis of emissions from a modern landfill site, for example, Camobreco *et al*. [140] used a timeframe of up to 500 years to allow for breakdown and release of the waste products, while in Switzerland, Doka and Hischier [141] extended analysis 60,000 years on the rationale that, by then, another ice age would have occurred, overwhelming the area! Such long timeframes may not only be difficult to rationalise in the more short-term world of policy-making, but also of course add to the uncertainties inherent in the analysis.

Nevertheless, in the face of dynamic and adaptive behaviours, static scenarios are clearly limited. While they may be useful in addressing general questions about the desirability of different policy goals, they give limited guidance on the likely consequences of trying to achieve these outcomes. In most cases, therefore, adaptive scenarios – or endogenous scenarios in the terminology of Carter and La Rovere [142] – are likely to be more informative. These do not define the ultimate state of the system, but instead specify the changes in input conditions; modelling is then done to simulate the way in which these move through the system, and the resulting system state. They are, as such, closer to projections or predictions than mere narratives or visions of some alternative world. Consequently, they are prey to all the inevitable uncertainties involved in modelling system behaviour. These uncertainties can be substantial in the case of environmental processes, where data are sparse, model parameterisation only approximate and non-linearity may rule. They are liable to become even greater, however, in the case of social systems that depend on human behaviours, for these are not always easy to predict and may seem to go against the purpose of the intervention or the collective good [7]. Adaptation, it needs to be remembered, is an intrinsic and largely individual phenomenon, and as such is each person's response to the perception of the world from within. As Hardin's [143] well-known parable of the 'tragedy of the commons' illustrates, from this perspective rational behaviour may look very different from that of the outside observer.

## Conclusion

The growing complexity of issues facing policy-makers, and the increasing demands for more inclusive and 'joined-up' policy have highlighted the need for more integrated methods of assessment to guide decision-taking. This need is especially acute in the area of environmental health, where the complexities of human activities, environmental processes, and human well-being come together. By extending the principles of integrated assessment, as previously developed mainly in the field of environmental policy, to human health much of this need can be addressed.

As this paper has indicated, however, the application of such integrated approaches to environmental health assessment brings many challenges. Chief among these are questions of how to deal properly with the multi-causality, non-linearity and change processes inherent in most analyses. Together these problems emphasise the need for careful and rigorous issue-framing and scenario specification as the foundation for assessment. How to achieve this, especially in the context of multiple stakeholders with varying interests and levels of expertise, is itself challenging. Conducting rigorous assessments of the scenarios thus defined presents further difficulties, not least because of gaps in data, limitations of knowledge and the inevitable amplification of uncertainties involved in devising and parameterising complex and linked models. These knowledge deficits, in turn, have important implications for the supporting sciences (especially of epidemiology and toxicology) – not least in demanding higher levels of understanding about the multivariate and time-varying interdependencies and interactions between environment and health. Many research challenges thus remain. If the larger problems that increasingly face society are to be resolved, however, these are all challenges that need to be taken up.

## Abbreviations

HEIMTSA: Health and Environment Integrated Methodology and Toolbox for Scenario Assessment (EU funded research project); INTARESE: Integrated Assessment of Health Risks from Environmental Stressors in Europe (EU funded research project); IPCS: Intergovernmental Panel on Climate Change; IRGC: International Risk Governance Council; REACH: Registration, Evaluation, Authorisation and Restriction of Chemical substances (EU Regulation); WHO: World Health Organisation.

## Competing interests

The author declares that he has no competing interests.

## Authors' contributions

This paper is the sole work of the author, who has been responsible for researching the issues covered, developing most of the new ideas and concepts contained herein, and preparing the paper.
